# Does complexity compromise outcomes in robotic hepatectomy?

**DOI:** 10.1007/s00464-025-12478-7

**Published:** 2025-12-17

**Authors:** Emrullah Birgin, Elisabeth Miller, Nadir Nasir, Leonard Bopp, Ali Kassem, Erik Rasbach, Moritz Schwab, Jan Heil, Dorothee Sturm, Marko Kornmann, Nuh N. Rahbari

**Affiliations:** Department of General and Visceral Surgery, Ulm University Hospital, Albert-Einstein-Allee 23, 89081 Ulm, Germany

**Keywords:** Difficulty, Liver resection, Minimally invasive liver surgery, DaVinci, Robotic surgery

## Abstract

**Background:**

Complex hepatectomies are challenging procedures in minimally invasive liver surgery. While the feasibility of complex laparoscopic hepatectomies is widely accepted, there is still limited data using the robotic platform for technically difficult cases. This cohort study evaluated postoperative outcomes in high and low complexity resections.

**Methods:**

Patients undergoing robotic hepatectomy recorded in a prospective database between 2020 and 2024 were included. Outcomes were compared between resections with a high complexity (HC) and low complexity (LC) based on the IWATE difficulty score system. Factors associated with severe morbidity were explored using logistic regression analyses.

**Results:**

Of 237 patients with a median age of 64 years (interquartile range 55–71), 125 patients had HC and 112 patients had LC resections. In the HC group, segmentectomies (*n* = 40, 32%) were the most frequently performed resections compared to non-anatomic hepatectomies (*n* = 97, 87%) in the LC group. The overall rate of postoperative morbidity was comparable between groups (comprehensive complication index 12.3 (standard deviation (SD) 21.6) vs. 7.5 (SD 17.6), *P* = 0.061), while operating time, blood loss, and rates of severe morbidity were higher in the HC group. There were no significant differences in achieving textbook outcome between groups (*n* = 88, 70% vs. *n* = 90, 80% *P* = 0.077). Major hepatectomy was identified as the sole independent risk factor for severe morbidity (OR 2.670, 95%CI 1.010—7.070, *P* = 0.048).

**Conclusion:**

Complex robotic hepatectomies are safe and feasible in high-volume centers with high rates of achieving textbook outcome.

**Graphic Abstract:**

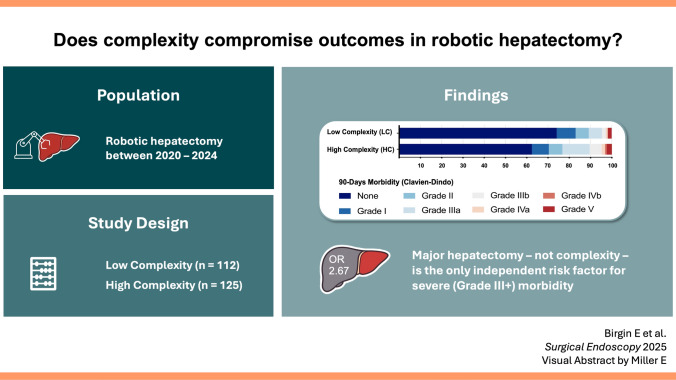

**Supplementary Information:**

The online version contains supplementary material available at 10.1007/s00464-025-12478-7.

Minimally invasive liver surgery has become increasingly adopted owing to improved perioperative and similar oncological outcomes compared to open hepatectomy [1–4].

While complex laparoscopic hepatectomies have been widely accepted in the literature, robotic surgery has emerged as an alternative technique. This platform may offer potential advantages, including enhanced dexterity, three-dimensional visualization, and improved ergonomics [5]. These features are particularly relevant in anatomically challenging resections, where robotic approaches have been associated with lower conversion rates, reduced blood loss, and improved perioperative outcomes compared with laparoscopy [6, 7]. Consequently, there is growing evidence that robotic hepatectomies may expand the feasibility of minimally invasive approaches to posterosuperior segments, major hepatectomies, and technically demanding parenchyma-sparing procedures [6, 8–10]. Despite these advances, the safety and outcomes of complex robotic hepatectomies remain insufficiently defined. Recently, a randomized trial comparing robotic and laparoscopic hepatectomies demonstrated no significant differences in perioperative outcomes; however, the trial primarily involved minor resections of variable complexity [11].

The IWATE score is widely used to grade hepatectomy complexity, but it was developed in the laparoscopic era [12] with some studies suggesting its applicability to robotic liver surgery [13–15]. While the scoring system ranges from 0 to 12 points, reflecting increasing technical complexity based on tumor characteristics (location, size, vascular proximity), extent of resection, Child–Pugh classification for liver function, and the use of hybrid or hand-assisted techniques, there is a persistent gap regarding outcomes of complex robotic hepatectomies. Accordingly, resections scoring 6 points or higher represent demanding resections including factors such as resections of the posterosuperior segments, anatomical hepatectomies, resections of larger tumors, and resections with proximity to major vessels—each of which may impact preoperative patient selection and potential risks and benefits of robotic hepatectomy. Therefore, the present study aimed to evaluate postoperative morbidity outcomes in patients undergoing complex robotic hepatectomies as defined by the IWATE score, and explore factors associated with severe morbidity.

## Methods

### Patients and study design

This single-center retrospective cohort study was based on data from a prospectively maintained institutional database and included all patients who underwent robotic liver resections for benign or malignant hepatic lesions at the University Hospital Ulm between November 2020 and December 2024. No formal sample size calculation was conducted; instead, all suitable patients treated during the specified timeframe were included. The study population comprised all consecutive adult patients (≥ 18 years) scheduled for liver resections. Patients with Klatskin tumors (perihilar cholangiocarcinoma), those who underwent biopsies only or portal vein ligation for staged hepatectomy, and those in whom no resection was ultimately performed were excluded from the analysis. This cohort study was reported in accordance with the STROBE (Strengthening the Reporting of Observational Studies in Epidemiology) statement [16]. The completed STROBE checklist is provided in the supplementary material.

### Surgical technique

All hepatectomies were performed using the da Vinci Xi robotic surgical system (Intuitive Surgical, Sunnyvale, CA, USA) as described previously [11, 17]. Patients were positioned in the reverse Trendelenburg position, and a pneumoperitoneum was established at a pressure of 12–18 mmHg [18]. Intraoperative ultrasound was performed in each case at the beginning of the procedure for resection guidance. The technique of parenchymal transection was determined intraoperatively at the surgeon’s discretion, utilizing either a robotic or a laparoscopic-assisted approach [19–23]. All surgeries were performed by experienced minimally invasive hepatobiliary surgeons [24]. Intrahepatic vessels and biliary structures were divided using either titanium clips (Braun) or Hem-o-lok clips (WECK). Major vascular pedicles and hepatic veins were divided using Endo Gia™ Stapler (Covidien) or the robotic stapling system (Intuitive). Vascular inflow control (Pringle maneuver) was used at the discretion of the operating surgeon during parenchymal transection; however, the application and duration were not recorded in a standardized manner. Abdominal drains were not routinely placed.

### Outcomes

Patient characteristics included age, gender, body mass index (BMI), American Society of Anesthesiologists (ASA) score classification, Charlson Comorbidity Index, cardiovascular comorbidities, diabetes and renal insufficiency. In addition, the histopathological presence of liver steatosis or cirrhosis and the recording of the Child–Pugh score. The extent of hepatectomy was based on the location of the liver lesion and surgeon’s discretion, ranging from a non-anatomic, partial hepatectomy to extended right or left hepatectomy, while a parenchyma-sparing approach was primarily intended. Liver resections were classified according to the Brisbane nomenclature and the updated Tokyo 2020 terminology [25, 26]. Anatomical liver resections were defined in line with Couinaud’s liver segmentation [26] and major hepatectomy was defined as the resection of more than three anatomical liver segments. Technical major resections were classified as anatomical hepatectomies or technically complex procedures including vessel exposures after parenchyma-sparing liver resections. Histopathological data were obtained from formal pathology reports. A negative resection margin (R0) was defined as a minimum clearance of 1 mm between tumor cells and the resection line. Postoperative morbidity was assessed using both the Comprehensive Complication Index [27] and the Clavien-Dindo classification [28], with severe complications defined as Clavien-Dindo grade IIIa or higher within 90 days of surgery. Posthepatectomy liver failure (PHLF), bile leakage (PHBL), and hemorrhage (PHH) were categorized and graded according to definitions established by the International Study Group of Liver Surgery (ISGLS), each classified into Grades A–C [29–31]. In brief, *Grade A* refers to abnormalities that do not require changes in management; *Grade B* involves noninvasive or nonoperative interventions; and *Grade C* calls for invasive procedures or surgical reintervention. Textbook outcome (TO) was defined based on seven intra- and postoperative quality indicators according to a consensus-based definition for hepatobiliary surgery [32].These include the absence of intraoperative incidents, no postoperative bile leakage or posthepatectomy liver failure (PHLF) of grade B or C, no major complications according to Clavien-Dindo grade IIIa or above, no 90-day readmission for surgical reasons, no 90-day mortality, and complete tumor resection. A patient is considered to have achieved a textbook outcome only if all seven criteria are met. The primary outcome was 90-day postoperative morbidity, compared between high and low complexity resections.

### IWATE scoring system

The IWATE scoring system was used to assess the level of technical complexity based on six criteria: tumor location, tumor size, proximity to major blood vessels, extent of hepatic resection, liver function according to the Child–Pugh classification, and the use of hybrid techniques [12]. Tumor location was assessed using the Couinaud segmentation system, with scores ranging from 1 point for segment III up to 5 points for segments VII and VIII. Tumor size was assigned 0 points for lesions smaller than 3 cm and 1 point for lesions measuring 3 cm or more. An additional point was added if the tumor was adjacent to major vessels, specifically, the main or second branch of the Glissonian tree, hepatic vein, or the inferior vena cava. Resection extent was scored as follows: 0 points for partial resection, 2 points for left lateral sectionectomy, 3 points for segmentectomy, and 4 points for sectionectomy or major hepatectomy. For hybrid approaches, intraoperative conversion led to a deduction of 1 point, whereas the absence of a hybrid technique resulted in 0 points. Accordingly, intraoperative conversion from robotic to open surgery was considered as a hybrid procedure. Liver function assessment allocated 0 points for Child–Pugh A classification and 1 point for Child–Pugh B. Preoperative imaging using computed tomography (CT), magnetic resonance imaging, and/or positron emission tomography–CT was performed to assess tumor location, size, and proximity to major vascular structures. In cases where multiple liver segments were equally affected, the segment associated with the highest level of surgical difficulty was selected for classification. High complexity (HC) liver resections were defined as hepatectomies scoring 6 points or higher on the IWATE classification. Resections scoring below 6 points were considered low complexity and were assigned to the LC group in line with a previous stratified randomized trial [11].

### Statistics

Descriptive statistics were presented as mean (standard deviation, SD) or median (interquartile range, IQR) for continuous variables with normal or non-normal distributions, and frequencies (percentage, %) for categorical variables. Categorical variables were compared using the Chi-Square test or Fisher’s exact test. The Mann–Whitney U test was used to analyze continuous variables. Univariate and multivariate logistic regression analyses were performed to evaluate associations between potential risk factors and clinically relevant postoperative complications (Clavien–Dindo ≥ IIIa) with adjustments for age, gender and Charlson Comorbidity Index. The selection of variables for the multivariate analysis was performed based on previous associations of risk factors with severe morbidity in literature and P-values < 0.2 on univariate analyses. Statistical significance was set at a P-value of < 0.05. All analyses were performed using R version 4.4.3 (Vienna, Austria).

## Results

### Patient characteristics

**Fig. 1:**
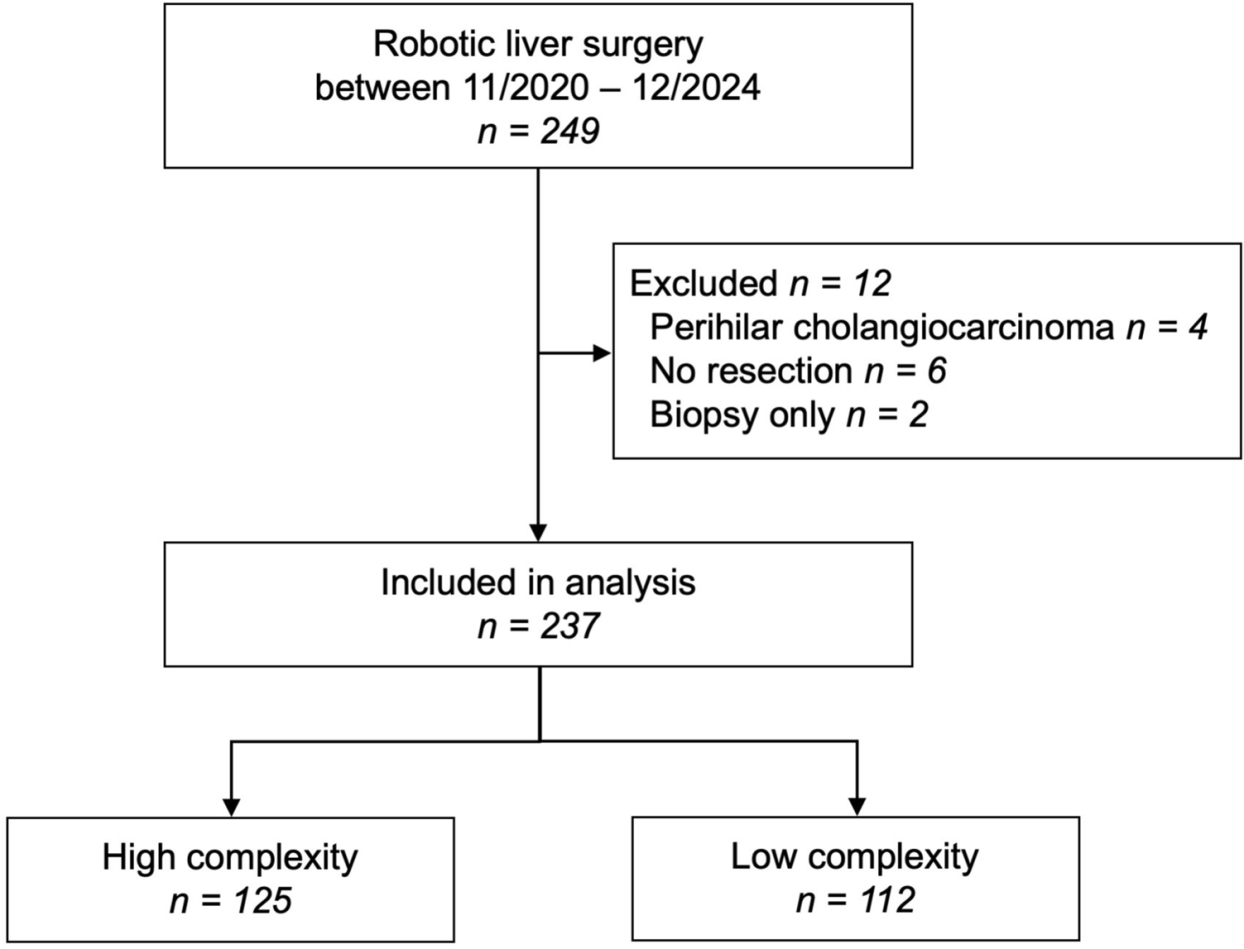
Study flow chart. High complexity (HC) liver resections were defined as hepatectomies scoring 6 points or higher on the IWATE classification. IWATE difficulty scoring system is based on extent of hepatic resection, tumor location, tumor size, proximity to major vessels, liver function, and hand-assisted hybrid procedures. Resections scoring below 6 points were considered low complexity (LC)

Of 249 robotic hepatectomies, 237 patients met the inclusion criteria (Fig. 1). Median age was 64 years (IQR 55–71) and median BMI was 26 kg/m^2^ (IQR 23–29) (Table 1). Cardiovascular comorbidities were present in 117 (49%) patients, while 47 (20%) had diabetes, and 17 (7%) had pre-existing renal insufficiency. A history of previous abdominal surgery was documented in 134 (57%) patients, including 27 (11%) with previous liver resections. Liver steatosis and cirrhosis were identified in 82 (35%) and 44 (19%) patients, respectively. Malignancies accounted for the majority of resections (n = 135, 57%), mainly hepatocellular carcinoma (n = 48, 20%) and liver metastases (n = 71, 30%). Among benign indications, alveolar echinococcosis was the predominant diagnosis (n = 52, 22%). Patients were stratified in the HC-group (n = 125, 53%) and LC-group (112, 47%). Baseline characteristics were similar, apart from a lower proportion of male patients in the HC-group (n = 59, 47% vs. n = 72, 64% P = 0.009).

**Table 1:** Baseline demographics of patients undergoing robotic liver resection, categorized into high- and low-complexity groups

Characteristics	Total n = 237	HC n = 125	LC n = 112	P-value
Age (years)	64 (55 – 71)	63 (55–71)	65 (56–71)	0.460
BMI (kg/m^2^)	26 (23 – 29)	26 (23–29)	26 (23–28)	0.443
Sex ratio, male:female	131:106	59:66	72:40	0.009
ASA				0.995
I/II	73 (31)	38 (30)	35 (31)	
III/IV	164 (69)	87 (70)	77 (69)	
Charlson comorbidity index	4 (2–4)	4 (2–6)	6 (2–6)	0.407
Cardiovascular comorbidities	117 (49)	63 (50)	54 (48)	0.795
Renal Insufficiency	17 (7)	10 (8)	7 (6)	0.626
Diabetes	47 (20)	25 (20)	22 (20)	< 0.999
Previous abdominal surgery	134 (57)	69 (55)	65 (58)	0.695
Previous liver surgery	27 (11)	18 (14)	9 (8)	0.153
Liver steatosis	82 (35)	47 (38)	35 (31)	0.340
Liver cirrhosis	44 (19)	25 (20)	19 (17)	0.617
Child–Pugh class				0.148
Child A	34 (77)	17 (68)	17 (90)	
Child B	10 (23)	8 (32)	2 (11)	
Diagnosis				0.092
HCC	48 (20)	31 (25)	17 (15)	
CCC	16 (7)	10 (8)	6 (5)	
Metastases	71 (30)	30 (24)	41 (27)	
Benign	102 (43)	54 (43)	48 (43)	
IWATE^a^ Score	6 (4–9)	9 (7–10)	3 (2–5)	< 0.001

Values are presented as median (interquartile range, IQR) or number (percentage), as appropriate. Statistical comparisons were made using the Mann–Whitney U test for continuous variables and the Chi-square or Fisher’s exact test for categorial variables

^a^IWATE difficulty scoring system is based on extent of resection, tumor location, tumor size, proximity to major vessels, liver function, and hand-assisted hybrid procedures

*HC*, high complexity; *LC*, low complexity; *ASA*, American Society of Anesthesiologists; *BMI*, body mass index; *HCC*, hepatocellular carcinoma; *CCC*, cholangiocellular carcinoma; *IQR*, interquartile range

### Surgical characteristics and complexity

**Fig. 2:**
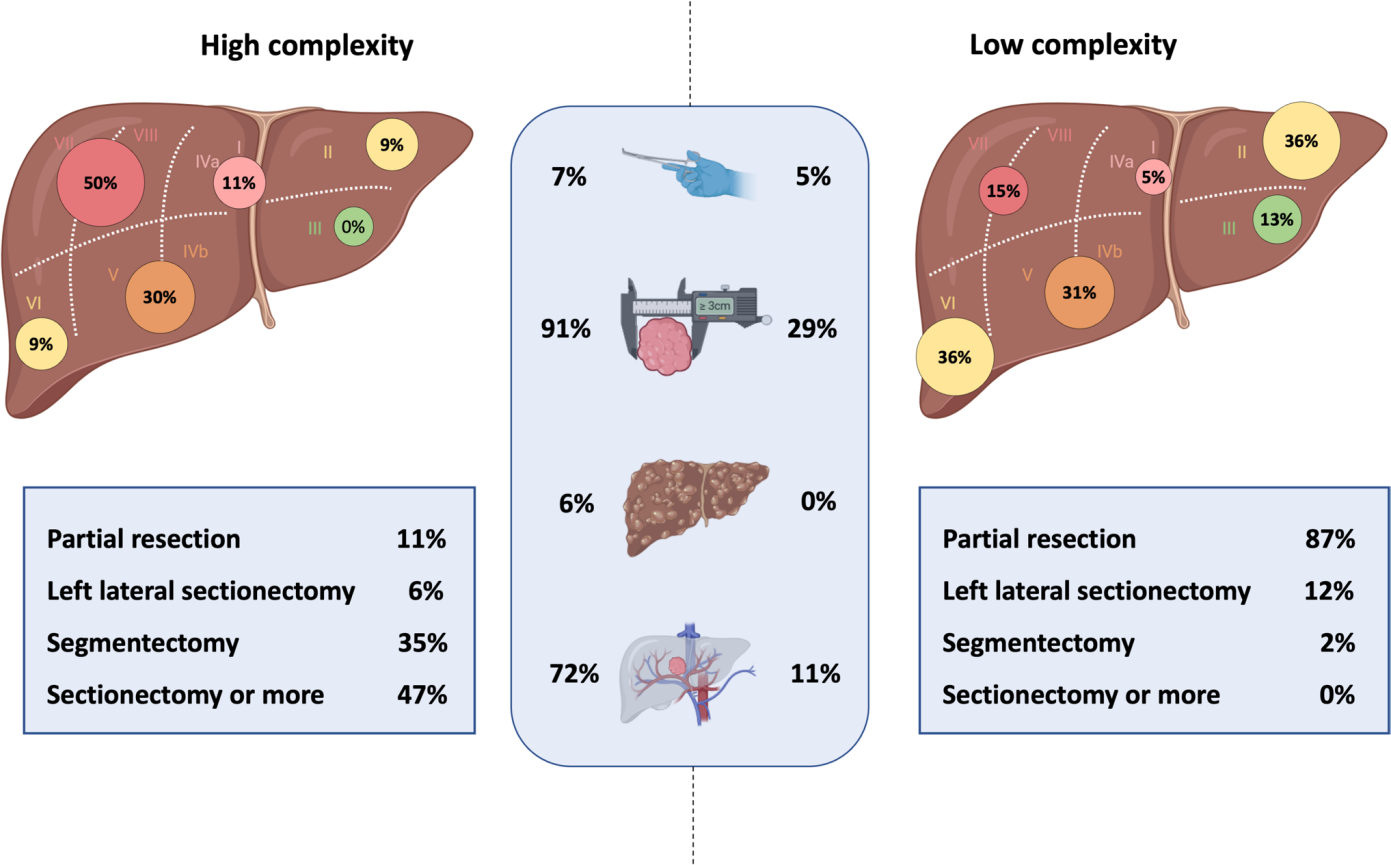
Resection complexity according to IWATE classification. Left panel, high-complexity resections; right panel, low-complexity resections. Liver illustrations indicate the proportion (%) of resections performed in each location, with color coding (green to red) showing increasing difficulty. Boxes directly beneath each diagram show the extent of resection within each complexity group. The central box summarizes additional key clinical variables: proportion of hybrid procedures, lesions larger than 3 cm, patients with Child B cirrhosis, and resections performed in proximity to major vessels. Percentages may not total 100% due to use of rounding to the nearest whole number

Tumors in the HC-group were more frequently ≥ 3 cm, vessel adjacent, and located in segments VII/VIII (Fig. 2). Conversion to open surgery was required in 14 patients (n = 9, 7% versus n = 5, 5%). According to the IWATE classification, "sectionectomies or more" procedures were the most frequent procedures in the HC-group, whereas none of LC-group patients fell within this category. Monosegmentectomies predominated in the HC-group (n = 40, 32%), while partial hepatectomies were most frequent in the LC-group (n = 97, 87%) (Table 2). Consequently, there were more technical major resections (n = 104, 83%). documented in the HC-group, while major hepatectomies were performed exclusively in the HC-group (n = 43, 34%). Median blood loss (400 mL vs. 250 mL, P < 0.001) and operative time (243 min vs. 164 min, P < 0.001) were significantly higher in the HC-group. R0 resection was achieved in 95% of cases overall, with no significant difference between the groups (94% vs. 96%, P = 0.513).

**Table 2:** Surgical characteristics of patients undergoing robotic liver resection categorized into high- and low-complexity groups

Characteristics	Total n = 237	HC n = 125	LC n = 112	*P*-value
**Extent of liver resection**				** < 0.001**
Major hepatectomy (> 3 segments)	43 (18)	43 (34)	0	
Minor hepatectomy (1–3 segments)	194 (82)	82 (66)	112 (100)	
*Main hepatectomy procedure*				
Non-anatomic, partial resection	111 (47)	14 (11)	97 (87)	
Segmentectomy (1 segment)	43 (18)	40 (32)	3 (3)	
Bisegmentectomy (2 segments)	38 (16)	26 (21)	12 (11)	
Multiple segments (> 2, e.g. central hepatectomy)	8 (3)	8 (6)	0 (0)	
(Extended) right hepatectomy	28 (12)	28 (22)	0(0)	
(Extended) left hepatectomy	9 (4)	9 (7)	0(0)	
**Technical major hepatectomy**	117 (49)	104 (83)	13 (12)	** < 0.001**
**Type of liver resection**				** < 0.001**
Anatomic	81 (34)	71 (57)	10 (9)	
Non-Anatomic	111 (47)	14 (11)	97 (87)	
Both	45(19)	40 (32)	5 (5)	
Blood loss (mL)	300 (200–600)	400 (250–700)	250 (100–425)	** < 0.001**
Operative time (min)	199 (146–275)	243 (180–326)	164 (132–211)	** < 0.001**
**R Status**				0.513
R0	224 (95)	117 (94)	107 (96)	
R1	13 (5)	8 (6)	5 (4)	

Values are presented as median (interquartile range, IQR) or number (percentage), as appropriate. Statistical comparisons were performed using the Mann–Whitney U test for continuous variables and the Chi-square or Fisher’s exact test for categorical variables. *HC*, high complexity; *LC*, low complexity; *IQR*, interquartile range; *R status*, resection margin status; *R0*, no residual tumor; *R1*, microscopic residual tumor

### Postoperative outcomes

**Fig. 3:**
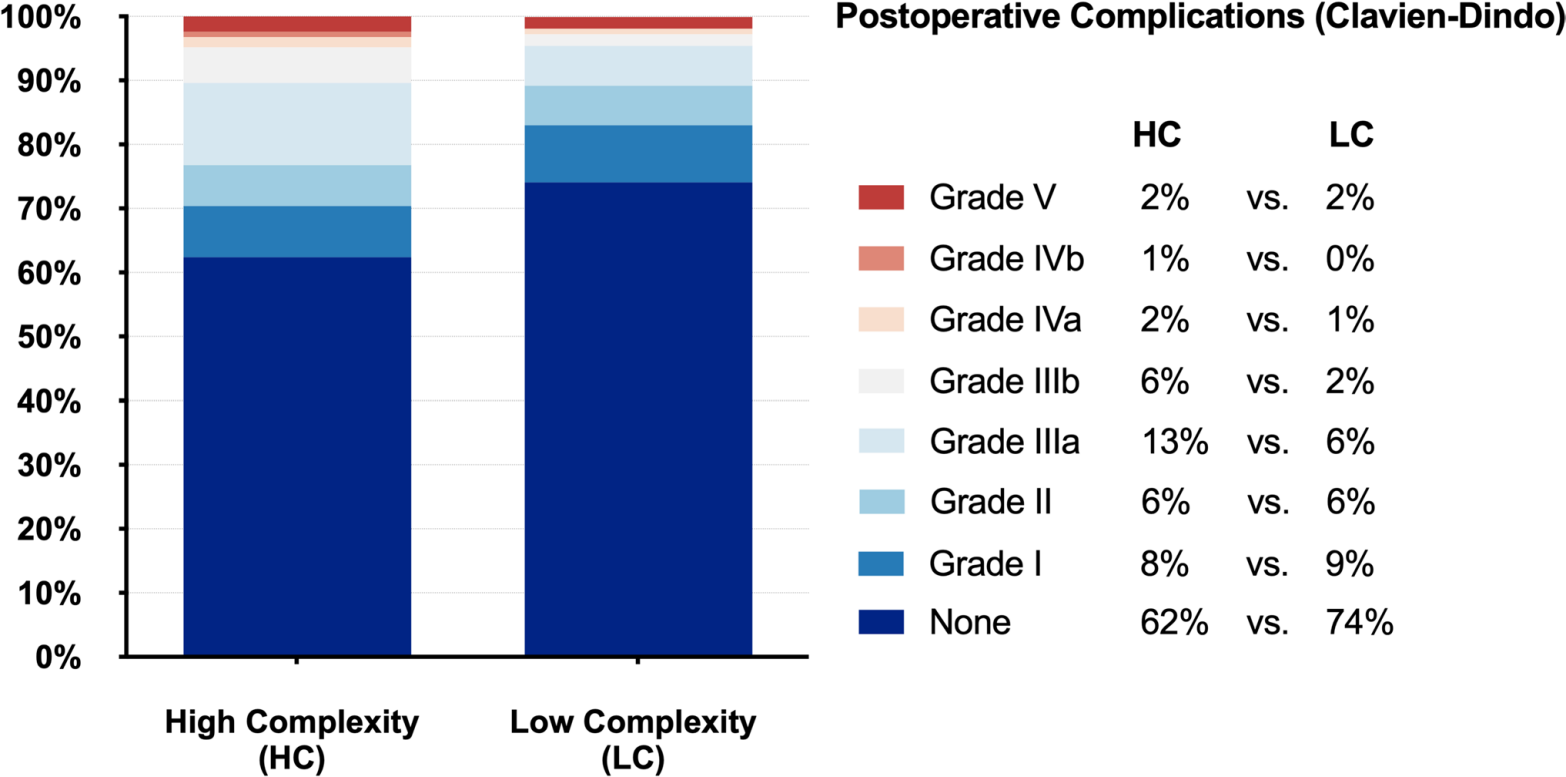
Frequency of postoperative complications according to Clavien–Dindo grade stratified by resection complexity. Frequency of postoperative complications according to Clavien–Dindo grade, stratified by resection complexity (High Complexity (HC) versus Low Complexity (LC)). Stacked bar charts display the distribution (%) of complications ranging from no complications to Clavien–Dindo grade V, separately for high- and low-complexity resections

Bile leakage was the most frequent complication (n = 15, 12% vs. n = 2, 2%, P = 0.009) (Table 3). Most leaks were managed by percutaneous drainage and, in cases with persistent leakage (> 200 mL/day for > 3 days) additional papillotomy was performed. Three patients with bile leakage required reoperation owing to failure of non-surgical measures: two underwent laparoscopic drainage of biliomas following central and non-anatomical resections, and one underwent laparotomy for a common bile duct defect. Posthepatectomy hemorrhage occurred in three patients, each receiving > 2 units of packed red blood cells; two required surgical revision to drain abdominal hematomas at the resection sites. PHLF was diagnosed in nine patients (eight grade B/C), predominantly after major hepatectomy or in cirrhosis. These patients received intravenous albumin and diuretics; some were transferred to intermediate or intensive care unit. Overall morbidity, assessed by the comprehensive complication index (12.3 (SD 21.6) vs. 7.5 (SD 17.6), P = 0.061) and Clavien-Dindo classification (P = 0.218, Fig. 3), did not differ significantly. However, severe complications were more frequent in the HC-group (n = 29, 23% vs. n = 12, 11%, P = 0.015). Hospital stay (6 vs. 5 days, P = 0.009) and unplanned readmissions (n = 13, 10% vs. n = 3, 3%, P = 0.020) were also higher in the HC-group. Five patients (2%) died within 90 days postoperatively: two from cardiac decompensation, two from PHLF in cirrhosis, and one from progressive cholangiocarcinoma. Textbook outcome was achieved in 75% overall (HC: n = 88, 70% vs. LC: n = 90, 80%, P = 0.077).

**Table 3:** Postoperative outcomes of patients undergoing robotic liver resection categorized into high- and low-complexity groups

Characteristics	Total n = 237	HC n = 125	LC n = 112	P-value
*Posthepatectomy complications*^*a*^				
Posthepatectomy bile leakage				0.009
Grade A	1 (0)	1 (1)	0 (0)	
Grade B	13 (6)	11 (9)	2 (2)	
Grade C	3 (1)	3 (2)	0 (0)	
*Posthepatectomy hemorrhage*				
Grade B	1 (0)	0 (0)	1 (1)	0.354
Grade C	2 (1)	2 (2)	0 (0)	
*Posthepatectomy liver failure*				
Grade A	1 (0)	1 (1)	0 (0)	0.521
Grade B	6 (3)	4 (3)	2 (2)	
Grade C	2 (1)	2 (2)	0 (0)	
Length of stay (days)	6 (4–8)	6 (5–9)	5 (4–8)	0.009
Readmission^b^	16 (7)	13 (10)	3 (3)	0.020
Textbook outcome^c^	178 (75)	88 (70)	90 (80)	0.077
Comprehensive Complication Index^d^	10.0 (19.9)	12.3 (21.6)	7.5 (17.6)	0.061

Values are presented as median (interquartile range, IQR), mean (standard deviation, SD) or number (percentage). Statistical comparisons were performed using the Mann–Whitney U test for continuous variables and the Chi-square or Fisher’s exact test for categorical variables

^a^Posthepatectomy complications as bile leakage, hemorrhage and liver failure are defined and graded according to the International Study Group of Liver Surgery (ISGLS) [29–31]. ^b^Unplanned Readmission within 90 days for surgical reasons. ^c^Textbook outcome is defined as the absence of intraoperative incidents, no postoperative bile leakage or posthepatectomy liver failure (PHLF) of grade B or C, no major complications according to Clavien-Dindo grade IIIa or above, no 90-day readmission for surgical reasons, no 90-day mortality, and complete tumor resection, ^d^Postoperative complications are graded according to the Comprehensive Complication Index (presented as mean) [27]. *HC*, high complexity; *LC*, low complexity; *IQR*, interquartile range; *SD*, standard deviation

### Risk factors for severe morbidity

As the likelihood of severe morbidity was higher in the HC-group, we further scrutinized potential perioperative risk factors associated with severe morbidity (Clavien Dindo ≥ IIIa) using univariate and multivariate logistic regression analyses (Supplementary Table 1). To account for potential imbalances in demographics such as sex differences, regression analyses were performed with adjustments for age, gender and Charlson Comorbidity Index. The multivariate analysis yielded that major hepatectomy was the only independent risk factor associated with a higher likelihood of severe morbidity (OR 2.670, P = 0.048), while complexity of resection was not associated with morbidity.

## Discussion

This study represents the largest single-center analysis to date evaluating morbidity and textbook outcome in complex robotic liver resections stratified by the IWATE system. We found that complex hepatectomies were associated with longer operative times, increased blood loss, and a higher rate of severe postoperative complications, particularly bile leakage. Despite these differences, length of stay was only marginally prolonged, and textbook outcome remained high at 75%, underscoring the overall safety and efficacy of robotic approaches in complex settings. The term *complex hepatectomy* is frequently used in the literature, yet a standardized definition remains lacking. In some reports, complexity is determined by the extent of resection, such as major hepatectomy involving three or more segments [33]. Others define complexity based on tumor location, particularly resections in posterosuperior segments or central liver regions, where exposure and parenchymal transection are technically demanding [34]. Still others emphasize parenchyma-sparing strategies such as monosegmentectomies, vascular or biliary reconstructions, or repeat resections [35] as hallmarks of complexity [36]. The absence of a uniform definition limits comparability across studies and hampers the development of validated benchmarks. Difficulty scoring systems such as the IWATE criteria provide structured stratification but were developed for laparoscopic approaches and may not fully capture the nuances of robotic surgery [12]. The robotic platform may offset some technical challenges emphasized in the IWATE classification system, such as posterosuperior resections, however, multiple other studies validated the use of the IWATE system in robotic surgery [37–39], including its adoption in the ROC’N’ROLL trial [11]. In the present study, segment VII/VIII resections and major hepatectomies were the predominant contributors to high complexity liver resections.

Our findings align with previous robotic series showing that IWATE-defined complexity correlates with intraoperative parameters but is less predictive of postoperative morbidity [13, 39]. Prior research indicated that operative time and blood loss increased across IWATE levels, yet postoperative complication rates remained similar between groups ranging from 7 to 36% [13, 39]. Notably, these studies involved relatively small cohorts (13–77 patients per complexity subgroup) and assessed postoperative outcomes within 30 days after surgery. In contrast, our study included 237 patients with 125 high-complexity cases, used an IWATE threshold of ≥ 6 to focus on technically demanding resections, and evaluated morbidity over 90 days, revealing complication rates of 26–38% across the study groups.

In our risk factor analysis, we found that reasons for severe morbidity were primarily due to major parenchyma loss, as major hepatectomy was the only independent variable. This finding is in line with other studies including major robotic hepatectomies with postoperative complication rates exceeding 30% [40]. This suggests that the IWATE score may underweight the impact of certain criteria within the scoring system [15]. The extent of parenchymal loss may have a disproportionately large effect on postoperative complications, while other criteria, such as tumor location, may pose technical challenges during surgery without translating to increased postoperative morbidity in robotic liver surgery. Thus, limitations of the IWATE score in robotic procedures should be considered, especially during case selection and surgical planning with careful evaluation of parenchymal loss.

In the present study, bile leakage was the most frequent complication after complex robotic hepatectomies which is consistent with previous reports [11, 41]. Although most bile leaks in our study were managed nonoperatively, these complications accounted for severe morbidity according to the Clavien-Dindo grading system. This finding reinforces the need for meticulous dissection and biliary management in complex hepatectomies. Other posthepatectomy-specific complications were comparable between the groups. Importantly, the oncological outcomes were not compromised in our study with reports of high rates of negative resection margins. Consequently, textbook outcome was achieved in three out of four patients despite cases of complex hepatectomies, which is higher than rates reported in the literature [42]. In addition, a benchmark study in a highly selective cohort identified the IWATE score and major hepatectomies as risk factors for not achieving textbook results [43]. A likely explanation for this finding is the higher morbidity rate associated with technically complex resections, which was also observed in our cohort. Notably, our rate of textbook outcome exceeded previous reported benchmarks [44] or was comparable to another study [43], supporting the role of robotic hepatectomy in experienced high-volume centers for technically demanding resections.

This study has limitations. First, the retrospective study design bears the risk of selection bias despite inclusion of consecutive patients. Second, the study reflects the experience of a high-volume, specialized center with experienced robotic liver surgeons, which may limit generalizability of results. Third, long-term oncological follow-up was beyond the scope of this analysis.

In conclusion, complex robotic hepatectomies are feasible in experienced hepatobiliary centers with low postoperative morbidity and high rates of textbook outcome. While complexity defined by the IWATE score correlated with increased perioperative burden, major hepatectomy was the key driver of severe morbidity. Future studies should refine robotic-specific risk stratification tools and validate these findings in multicenter, prospective cohorts to guide patient selection, training, and standardization of robotic liver surgery.

## Supplementary Information

Below is the link to the electronic supplementary material.

Supplementary file1 (DOCX 30 KB)

## Data Availability

The datasets generated and analyzed during the current study are available from the corresponding author on reasonable request.
