# Supplementary material for: Does complexity compromise outcomes in robotic hepatectomy?
Source: Surg Endosc. 2025 Dec 17;40(3):2142–52. doi: 10.1007/s00464-025-12478-7 (PMC12971833; doi:10.1007/s00464-025-12478-7)
Supplement: Supplementary file 1 — Supplementary file1 (DOCX 30 KB) [file 464_2025_12478_MOESM1_ESM.docx]

**SUPPLEMENTALS**

**Supplementary Table 1:** Univariate and Multivariate Logistic Regression Analyses of of Risk Factors for Severe Complications

|  | **Univariate Analysis** | | |  | **Multivariate Analysis** | | |
| --- | --- | --- | --- | --- | --- | --- | --- |
| **Variable** | **OR** | **95% - CI** | **P-value** |  | **OR** | **95% - CI** | **P-value** |
| **Complexity** |  |  |  |  |  |  |  |
| Low | Ref |  |  |  | Ref |  |  |
| High | 2.840 | 1.330 – 6.050 | 0.001 |  | 1.620 | 0.525 – 4.990 | 0.402 |
| **Technical major resection** |  |  |  |  |  |  |  |
| No | Ref |  |  |  | Ref |  |  |
| Yes | 2.640 | 1.290 – 5.410 | 0.008 |  | 1.140 | 0.378 – 3.430 | 0.817 |
| **ASA** |  |  |  |  |  |  |  |
| I/II | Ref |  |  |  | Ref |  |  |
| III/IV | 2.640 | 1.050 – 6.600 | 0.038 |  | 1.100 | 0.352 – 3.410 | 0.875 |
| **Cardiac comorbidity** |  |  |  |  |  |  |  |
| Absent | Ref |  |  |  | Ref |  |  |
| Present | 2.590 | 1.270 – 5.300 | 0.009 |  | 1.650 | 0.689 – 3.930 | 0.261 |
| **Renal insufficiency** |  |  |  |  |  |  |  |
| Absent | Ref |  |  |  | Ref |  |  |
| Present | 2.130 | 0.707 – 6.420 | 0.179 |  | 1.700 | 0.488 – 5.920 | 0.405 |
| **Previous liver surgery** |  |  |  |  |  |  |  |
| No | Ref |  |  |  | Ref |  |  |
| Yes | 2.260 | 0.913 – 5.590 | 0.007 |  | 1.310 | 0.457 – 3.740 | 0.617 |
| **Liver cirrhosis** |  |  |  |  |  |  |  |
| No | Ref |  |  |  | Ref |  |  |
| Yes | 2.120 | 0.980 – 4.590 | 0.056 |  | 2.750 | 0.745 – 10.100 | 0.129 |
| **Diagnosis** |  |  |  |  |  |  |  |
| HCC | Ref |  |  |  | Ref |  |  |
| CCC | 1.360 | 0.394 – 4.730 | 0.625 |  | 1.880 | 0.457 – 7.700 | 0.383 |
| Metastases | 0.672 | 0.277 – 1.630 | 0.381 |  | 2.260 | 0.553 – 9.250 | 0.256 |
| Benign | 0.363 | 0.147 – 0.896 | 0.028 |  | 1.010 | 0.261 – 3.920 | 0.986 |
| **Extent of liver resection** |  |  |  |  |  |  |  |
| Minor hepatectomy | Ref |  |  |  | Ref |  |  |
| Major hepatectomy | 3.460 | 1.630 – 7.340 | 0.001 |  | 2.670 | 1.010 – 7.070 | **0.048** |

Adjustments for age, gender and Charlson Comorbidity Index was performed.

*OR=odds ratio, CI=confidence interval, Ref = Reference, ASA*=American Society of Anesthesiologists*, HCC*=hepatocellular carcinoma; *CCC*=cholangiocellular carcinoma; *CRLM*=colorectal liver metastases

**Supplementary File: STROBE Checklist**

STROBE Statement—checklist of items that should be included in reports of observational studies

|  | Item No. | Recommendation | Page  No. | Relevant text from manuscript |
| --- | --- | --- | --- | --- |
| **Title and abstract** | 1 | (*a*) Indicate the study’s design with a commonly used term in the title or the abstract | 2 | “This Cohort Study” (p. 2, l. 6) |
|  |  | (*b*) Provide in the abstract an informative and balanced summary of what was done and what was found | 2 | See “Abstract” (p. 2, l. 1-30) |
| Introduction | | | |  |
| Background/rationale | 2 | Explain the scientific background and rationale for the investigation being reported | 3 | “Introduction” (p. 3, l. 1-28) |
| Objectives | 3 | State specific objectives, including any prespecified hypotheses | 3 | “…the present study aimed to evaluate postoperative morbidity outcomes …” (p. 3, l. 29-31) |
| Methods | | | |  |
| Study design | 4 | Present key elements of study design early in the paper | 4 | “This single-center retrospective cohort study…” (p. 4, l. 3-6) |
| Setting | 5 | Describe the setting, locations, and relevant dates, including periods of recruitment, exposure, follow-up, and data collection | 4,5 | “…based on data from a prospectively maintained institutional database and included all patients who underwent robotic liver resections for benign or malignant hepatic lesions at the University Hospital Ulm between November 2020 and December 2024.” (p. 4, l. 3-6). “Postoperative morbidity was assessed… within 90 days of surgery.” (p. 5, l. 17-20) |
| Participants | 6 | (*a*) *Cohort study*—Give the eligibility criteria, and the sources and methods of selection of participants. Describe methods of follow-up  *Case-control study*—Give the eligibility criteria, and the sources and methods of case ascertainment and control selection. Give the rationale for the choice of cases and controls  *Cross-sectional study*—Give the eligibility criteria, and the sources and methods of selection of participants | 4,5 | “The study population comprised all consecutive adult patients… and those in whom no resection was ultimately performed were excluded from the analysis.” (p. 4, l.9-12) |
|  |  | (*b*) *Cohort study*—For matched studies, give matching criteria and number of exposed and unexposed  *Case-control study*—For matched studies, give matching criteria and the number of controls per case |  |  |
| Variables | 7 | Clearly define all outcomes, exposures, predictors, potential confounders, and effect modifiers. Give diagnostic criteria, if applicable | 5 | see Methods – *Outcomes (p. 5, l. 2-34)* |
| Data sources/ measurement | 8* | For each variable of interest, give sources of data and details of methods of assessment (measurement). Describe comparability of assessment methods if there is more than one group | *6* | *(p. 6, l. 2-24)* |
| Bias | 9 | Describe any efforts to address potential sources of bias | 10 | “…regression analyses were performed with adjustments for age, gender and Charlson Comorbidity Index.” (p.9, l. 22-24) |
| Study size | 10 | Explain how the study size was arrived at | 4 | “No formal sample size calculation was conducted; instead, all suitable patients treated during the specified timeframe were included.” (p. 4, l. 6-8) |

| Quantitative variables | 11 | Explain how quantitative variables were handled in the analyses. If applicable, describe which groupings were chosen and why | 6 | “High complexity (HC) liver resections were defined as hepatectomies scoring 6 points or higher on the IWATE classification. Resections scoring below 6 points were considered low complexity and were assigned to the LC group in line with a previous stratified randomized trial…” (p. 6, l. 21-24) |
| --- | --- | --- | --- | --- |
| Statistical methods | 12 | (*a*) Describe all statistical methods, including those used to control for confounding | 6,7 | See Methods – *Statistics (p. 6, l. 27-34, p. 7, l. 1-4)* |
|  |  | (*b*) Describe any methods used to examine subgroups and interactions | 6,7 | See Methods – *Statistics (p. 6, l. 29-34, p. 7, l. 1-4)* |
|  |  | (*c*) Explain how missing data were addressed | - |  |
|  |  | (*d*) *Cohort study*—If applicable, explain how loss to follow-up was addressed  *Case-control study*—If applicable, explain how matching of cases and controls was addressed  *Cross-sectional study*—If applicable, describe analytical methods taking account of sampling strategy | - |  |
|  |  | (*e*) Describe any sensitivity analyses | 6,7 | See Methods - *Statistics* |
| Results | | | | |
| Participants | 13* | (a) Report numbers of individuals at each stage of study—eg numbers potentially eligible, examined for eligibility, confirmed eligible, included in the study, completing follow-up, and analysed | 8 | “Of 249 robotic hepatectomies, 237 patients met…” (p. 8, l. 3) |
|  |  | (b) Give reasons for non-participation at each stage | 4  Flow Chart | “…were excluded from the analysis.” (p. 4, l. 9-12) |
|  |  | (c) Consider use of a flow diagram | Flow Chart | See Flow Chart |
| Descriptive data | 14* | (a) Give characteristics of study participants (eg demographic, clinical, social) and information on exposures and potential confounders | 8 | “Median age was 64 years …” (p. 8, l. 3-14), Table 1 |
|  |  | (b) Indicate number of participants with missing data for each variable of interest | - |  |
|  |  | (c) *Cohort study*—Summarise follow-up time (eg, average and total amount) | 8 | “…within 90 days of postoperatively…” (p. 8, l. 13) |
| Outcome data | 15* | *Cohort study*—Report numbers of outcome events or summary measures over time | *9* | “Overall morbidity…” (p. 9, l. 8-10) Table 3 |
|  |  | *Case-control study—*Report numbers in each exposure category, or summary measures of exposure | *-* |  |
|  |  | *Cross-sectional study—*Report numbers of outcome events or summary measures | *-* |  |
| Main results | 16 | (*a*) Give unadjusted estimates and, if applicable, confounder-adjusted estimates and their precision (eg, 95% confidence interval). Make clear which confounders were adjusted for and why they were included | 9 | “Overall morbidity…” (p. 9, l. 8-10), “…we further scrutinized potential perioperative risk factors associated with severe morbidity (Clavien Dindo ≥IIIa) using univariate and multivariate logistic regression analyses” (p. 9, l. 19-21) |
|  |  | (*b*) Report category boundaries when continuous variables were categorized | 4 | Methods – *Outcomes (p.4, l. 2-34)* |
|  |  | (*c*) If relevant, consider translating estimates of relative risk into absolute risk for a meaningful time period |  |  |

| Other analyses | 17 | Report other analyses done—eg analyses of subgroups and interactions, and sensitivity analyses | 9 | (p. 9, l. 19-27) |
| --- | --- | --- | --- | --- |
| Discussion | | | | |
| Key results | 18 | Summarise key results with reference to study objectives | 11 | “…we found that reasons for severe morbidity were primarily due to major parenchyma loss…” (p. 11, l. 3-4) |
| Limitations | 19 | Discuss limitations of the study, taking into account sources of potential bias or imprecision. Discuss both direction and magnitude of any potential bias | 11 | “This study has limitations…” (p. 11, l. 32) |
| Interpretation | 20 | Give a cautious overall interpretation of results considering objectives, limitations, multiplicity of analyses, results from similar studies, and other relevant evidence | 12 | “Future studies should refine robotic-specific risk stratification tools and validate these findings in multicenter, prospective cohorts to guide patient selection, training, and standardization of robotic liver surgery…” (p. 12, l. 6-9) |
| Generalisability | 21 | Discuss the generalisability (external validity) of the study results | 11, 12 | “…experience of a high-volume.. which may limit generalizability…” (p. 11, l. 33-34; p. 12, l. 1) |
| Other information | |  | | |
| Funding | 22 | Give the source of funding and the role of the funders for the present study and, if applicable, for the original study on which the present article is based | 1 | “This research did not receive any specific grants from…” |

*Give information separately for cases and controls in case-control studies and, if applicable, for exposed and unexposed groups in cohort and cross-sectional studies.

**Note:** An Explanation and Elaboration article discusses each checklist item and gives methodological background and published examples of transparent reporting. The STROBE checklist is best used in conjunction with this article (freely available on the Web sites of PLoS Medicine at http://www.plosmedicine.org/, Annals of Internal Medicine at http://www.annals.org/, and Epidemiology at http://www.epidem.com/). Information on the STROBE Initiative is available at www.strobe-statement.org.
